# Analysis of the spatial and temporal arrangement of transcripts over intergenic regions in the human malarial parasite *Plasmodium falciparum*

**DOI:** 10.1186/1471-2164-14-267

**Published:** 2013-04-19

**Authors:** Karen Russell, Sandra Hasenkamp, Richard Emes, Paul Horrocks

**Affiliations:** 1 Institute for Science and Technology in Medicine, Keele University, Huxley Building, Staffordshire ST5 5BG, United Kingdom; 2 School of Veterinary Medicine and Science, University of Nottingham, Sutton Bonington, Leicestershire LE12 5RD, United Kingdom

**Keywords:** Malaria, Apicomplexan parasites, Gene organisation, Regulation of gene expression

## Abstract

**Background:**

The ability of the human malarial parasite *Plasmodium falciparum* to invade, colonise and multiply within diverse host environments, as well as to manifest its virulence within the human host, are activities tightly linked to the temporal and spatial control of gene expression. Yet, despite the wealth of high throughput transcriptomic data available for this organism there is very little information regarding the location of key transcriptional landmarks or their associated *cis*-acting regulatory elements. Here we provide a systematic exploration of the size and organisation of transcripts within intergenic regions to yield surrogate information regarding transcriptional landmarks, and to also explore the spatial and temporal organisation of transcripts over these poorly characterised genomic regions.

**Results:**

Utilising the transcript data for a cohort of 105 genes we demonstrate that the untranscribed regions of mRNA are large and apportioned predominantly to the 5′ end of the open reading frame. Given the relatively compact size of the *P*. *falciparum* genome, we suggest that whilst transcriptional units are likely to spatially overlap, temporal co-transcription of adjacent transcriptional units is actually limited. Critically, the size of intergenic regions is directly dependent on the orientation of the two transcriptional units arrayed over them, an observation we extend to an analysis of the complete sequences of twelve additional organisms that share moderately compact genomes.

**Conclusions:**

Our study provides a theoretical framework that extends our current understanding of the transcriptional landscape across the *P*. *falciparum* genome. Demonstration of a consensus gene-spacing rule that is shared between *P*. *falciparum* and ten other moderately compact genomes of apicomplexan parasites reveals the potential for our findings to have a wider impact across a phylum that contains many organisms important to human and veterinary health.

## Background

*Plasmodium falciparum*, the aetiological agent of the most severe form of human malaria, imposes a significant health and socioeconomic impact on those regions of the world where this parasite is endemic [1]. This malarial parasite has a lifecycle that alternates between a human host and mosquito vector, requiring multiple morphological and biological adaptations to successfully invade, colonise and divide within diverse cellular environments. Progression of parasites through this complex life cycle and the manifestation of virulence within the human host are both tightly linked to the temporal and spatial control of gene expression [2-9]. Over recent years we have garnered a greater appreciation of the interplay between the molecular mechanisms operating at the genetic and epigenetic levels in regulating developmentally-linked gene expression [4-6,8]. These insights have been provided by global analyses of the temporal programme of steady-state transcript accumulation [10-12], mRNA stability and RNA polymerase II complex activity [13-16]. Yet despite these advances, and with access to a fully-annotated genome [17], we know relatively little regarding the fundamental organisation of the transcriptional unit in this important pathogen. This bottleneck arises from the extreme AT nucleotide bias in the intergenic regions (IGR). Here AT content typically exceeds 80-90%, imposing significant challenges for amplifying, cloning and sequencing of these regions as well as the application of bioinformatics tools (e.g. the unambiguous mapping of sequence reads from massive parallel sequencing of cDNA). Thus, we understand very little regarding the nature of the transcriptional unit outside of the open reading frame (ORF).

Determining the coordinates of the transcriptional start and stop sites is important. Sequences adjacent to transcriptional start sites likely comprise the *cis*-acting elements to which the regulatory and basal components of the RNA polymerase II complex bind. Moreover, these coordinates identify sequences in the 5′ and 3′ untranslated regions (UTR) of the transcript. These UTR similarly contain *cis*-acting sequences that direct translational efficiency, mRNA capping and stability. Knowing the number and position of transcription start sites in *P*. *falciparum* is potentially important as it may provide key clues to the different molecular mechanisms employed in the control of transcription. For example, is there a generally relaxed transcriptional activation process that relies on molecular mechanisms downstream to regulate temporal patterns of steady-state transcript accumulation? This model is certainly supported by recent reports of a global programme of temporal mRNA stability during intraerythrocytic development [14]. Or, does the parasite utilise a single predominant transcription start site that employs specific *cis*–*trans* interactions over a core promoter to drive temporal expression? This was not previously a favoured model given the apparent paucity of specific transcription factors in the parasite’s genome [18-20], but it has recently regained support following the identification and characterisation of an expanded family of novel specific transcription factors (ApiAP2) in apicomplexan parasites [21-26]. A combination of both models is likely at play – but resolving the issue of where these key transcriptional coordinates are located is essential.

**Figure 1:**
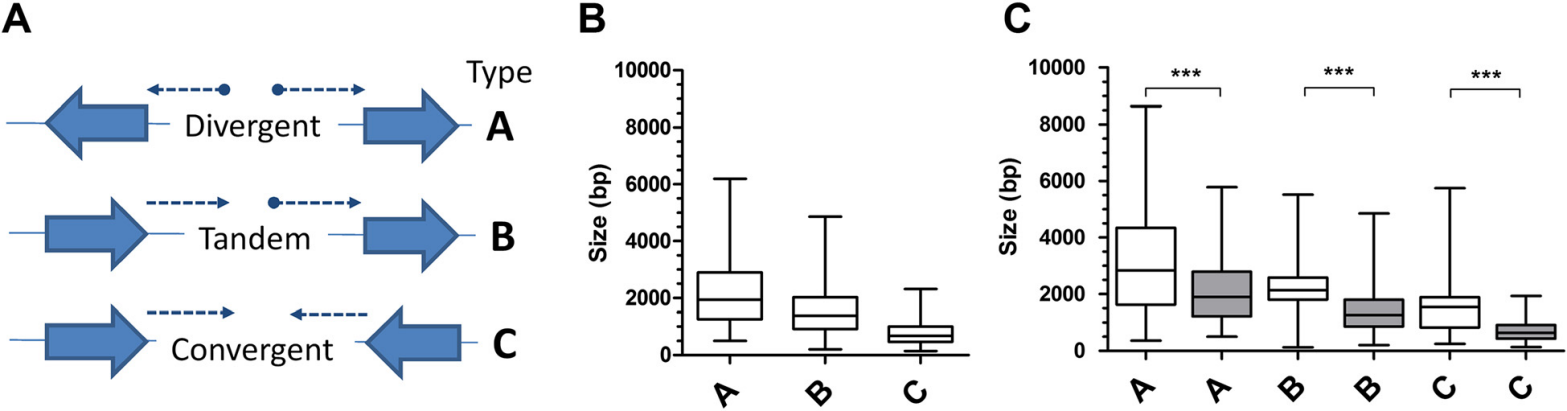
**Distribution of IGR size in *P. falciparum*. A**) Schematic representing the orientation of divergent, tandem and convergent transcriptional units over IGR types A, B and C, respectively. Block arrows represent the orientation of flanking ORF, transcripts are indicated as dotted lines with the direction of transcription indicated using an arrowhead. Where relevant, the 5′ end of a transcript is indicated with a solid filled dot. For simplicity, only non-overlapping transcriptional units are represented. **B**) Box and whisker plot representing the distribution of size of IGR types A, B and C. The box represents the 25-75% distribution, the enclosed line the median, with the whiskers indicating the range of sizes between 2.5-97.5% of the entire range. Due to the distribution of data, outliers beyond the 2.5-97.5% of data represented by the range whiskers are not shown. **C**) Box and whisker plots representing the distribution of the size of IGR types A, B and C in subtelomeric (clear boxes) and chromosomal internal (grey shaded boxes) domains. For each pair of IGR type, the differences are significant (ANOVA, *** p < 0.001). Due to the distribution of data, outliers beyond the 2.5-97.5% of data represented by the range whiskers are not shown.

Studies on the size and organization of IGR in fungal species, which share a similarly compact genome as *P*. *falciparum*, suggest that transcriptional and RNA processing *cis*-acting regulatory sequences leave a “footprint” on the IGR [27,28]. IGR that contain divergent transcripts, i.e. the flanking open reading frames (ORF) are orientated in a head-to-head fashion (see Figure 1A), are larger than those IGR with convergent transcriptional units where the flanking ORF are organised tail-to-tail. These studies indicate that gene spacing is not random, but is instead organised to facilitate the spatial arrangement of transcriptional units, and also that 5′ UTR are larger than 3′ UTR. A provisional analysis of IGR spaces from the incomplete chromosome 3 of *P*. *falciparum* indicates the same gene spacing patterning is present [29]. However, to date, no studies have addressed the spatial and temporal organisation of transcripts over these IGR.

As indicated above, there is a critical lack of data concerning the *P*. *falciparum* transcriptional unit outside of the ORF. Expressed sequence tag (EST) data from 3′ rapid amplification of cDNA ends (3′ RACE) and RNA ligase mediated RACE (RLM-RACE) provide some coverage. For example, RLM-RACE provides transcription start data for 1465 ORF (c. 27% of total) and is available through the Full-Malaria database (http://fullmal.hgc.jp) [30,31]. These data indicate that *P*. *falciparum* transcriptional start sites are generally located at multiple loci, often spread over several hundred basepairs, some 150-450 bp upstream of the ORF. In addition to these genomic approaches, there are also a number of single-gene studies that provide transcript size data from Northern blots (see Additional file 1 and Additional file 2). Whilst many of these studies do not report the physical mapping of transcriptional start and stop sites, they do generally indicate two features of the *P*. *falciparum* transcript that seem at odds with the available EST data. First, transcripts are typically much larger than the ORF, suggesting a significant fraction of a transcript is untranslated. Second, one or two major transcripts are most often observed, which would suggest either that only one or two major transcription start sites exist, or that if many transcription start sites are utilised then these are either very close together or else only one or two give rise to a major stable transcript. Assays of promoter structure that are complemented with physical mapping of the transcription start site suggest that transcripts initiate at one, or at two closely located, transcription start sites and that these extend between 400-1900 bp upstream of the ORF [5,32-37]. Despite what appears to be a disparity between the size of UTR predicted from EST and Northern blot studies, no systematic comparison of these data has been carried out to date to explore this difference.

We describe here a study that explores the size and organisation of IGR in *P*. *falciparum* and correlates this with UTR data available from Northern blots and EST databases. Our findings suggest that *P*. *falciparum* transcripts have a large UTR which appears preferentially apportioned to the 5′ end of the ORF. As this would suggest that significant amounts of the IGR that flank ORF are included in transcripts, we explore how transcriptional units are spatially and temporally organised over these IGR. Further, by showing a similar IGR arrangement in other apicomplexan parasites important for human and animal health, we suggest that our findings may impact more widely in understanding the molecular control of transcription across this phylum.

## Results

### The size of IGR is related to the transcriptional activity that occurs within that space

The sizes of all 5588 IGR in *P*. *falciparum* (clone 3D7) were determined and categorised into one of three groups (A, B or C) to reflect the nature of transcriptional activity that occurs over them (Figure 1A). Group A IGR contain two divergent transcripts, orientated towards the flanking head-to-head ORF and thus contain two promoters (two 5′ UTR). Group B IGR contain two tandem arrayed transcripts over the head-to-tail flanking ORF with one promoter (5′ UTR) and one terminator (3′ UTR). The remaining type C IGR contain two convergent transcripts over the flanking tail-to-tail ORF and two terminators (two 3′ UTR). There are 1479, 2626 and 1483 of types A, B and C IGR, respectively, which gives a relative ratio of 1:1.77:1 (Table 1), close to the expected 1:2:1 ratio expected from the known organization of *P*. *falciparum* genes into monocistronic transcriptional units [5,38,39]. The sizes of IGR in the three groups are significantly different (Figure 1B, p < 0.05) showing the relationship A > B > C (medians of 1938, 1385 and 677 bp, respectively) as a 2.9:2:1 ratio. Thus, IGR size in *P*. *falciparum* clearly correlates with the orientation of transcriptional units arrayed over them with 5′ flanking IGR generally larger than 3′ flanking IGR.

**Table 1:** **IGR size and distribution in *P. falciparum ***

**Region**	**IGR Type**	**n=**	**Ratio of IGR types**^**1**^	**Median size (bp)**	**% change**^**2**^	**Ratio of median size**^**1**^
All genome	A	1479	1.00	1938		2.86
	B	2626	1.77	1385		2.05
	C	1483	1.00	677		1.00
Subtelomeric	A	123	0.98	2838	+46.4	1.84
	B	379	3.03	2138	+54.4	1.38
	C	125	1.00	1545	+128.2	1.00
Internal	A	1283	1.01	1905	−1.1	2.95
	B	2118	1.66	1266	−8.6	1.96
	C	1276	1.00	646	−4.6	1.00

^1^ Type C IGR is always defined as 1.00. ^2^% change compared to data from IGR in all genome.

*P*. *falciparum* chromosomes are typically divided into subtelomeric and chromosome-internal domains; reflecting their differing heterochromatic environment, multigene family composition, sub-nuclear organization and length plasticity [3,9,40-45]. Whilst we know there is a reduced gene density within subtelomeric regions, whether this is reflected in differences in the size and orientation of IGR is not known. We determined the 28 breakpoints between the subtelomeric/chromosome-internal regions for the 14 chromosomes of *P*. *falciparum* (Additional file 3) based on the loss of synteny with the related *Plasmodium spp*. *P*. *knowlesi* and *P*. *vivax*. 627 IGR (11.8% of total) were defined as falling within the subtelomeric region. The ratio of types A, B and C IGR in the subtelomeric region is approximately 1:3:1 (123:379:125) (Table 1), reflecting the known bias for head-to-tail orientation of the numerous members of the *rifin* multi-gene family present in this region [46]. Subtelomeric IGR, however, were all significantly larger (p < 0.05) than those in the chromosome internal regions (Figure 1C). This increase in size was not equitable across the different classes of IGR (Table 1), resulting in an alteration of the A:B:C IGR spacing ratio from approximately 3:2:1 to 1.8:1.4:1.

A preliminary analysis on the sizes of IGR from chromosome 3 of *P*. *falciparum* reported that A > B > C and that they show a relative 3:1.9:1 size ratio; close to that reported here (2.9:2:1) for the entire genome [29]. This study also describes an analysis of the partial genome of the similarly AT-rich organism *Dictyostelium discoideum*, and concluded that a 3:2:1 length ratio for IGR types A, B and C appears to be broadly true across moderately compact genomes (2.5-4.8 Kb/ORF). We extended this preliminary analysis to encompass the entire genomes of *D*. *discoideum*, the yeast *Saccharomyces cerevisiae*, and ten additional apicomplexan parasites (*P*. *knowlesi*, *P*. *vivax*, *P*. *yoelii*, *Babseia bovis*, *Cryptosporidium hominis*, *C*. *parvum*, *Neospora caninum*, *Toxoplasma gondii*, *Theileria annulata and T*. *parva*[47-55]) that exhibit a range of AT content and genome density (Table 2) to determine whether this orientation-specific effect on IGR length held true on wider investigation.

**Table 2:** Comparison of the size and organism of IGR from organisms used in this study

**Organism**		**IGR count**			**Ratio of IGR count**^**2**^			**Median size of IGR (bp)**			**Ratio of median size**^**2**^			**Significant difference**^**3**^		
	**% AT**^**1**^	**A**	**B**	**C**	**A**	**B**	**C**	**A**	**B**	**C**	**A**	**B**	**C**	**AvB**	**AvC**	**BvC**
*Babesia bovis*	58.2	1124	1990	1032	1.1	1.9	1.0	543	352	175	3.1	2.0	1.0	Yes	Yes	Yes
*Crytosporidium hominis*	68.3	328	631	404	0.8	1.6	1.0	640	494	203	3.2	2.4	1.0	Yes	Yes	Yes
*Crytosporidium parvum*	70	994	1666	972	1.0	1.7	1.0	634	460	175	3.6	2.6	1.0	Yes	Yes	Yes
*Dictyostelium discoideum*	77.6	3312	6571	3313	1.0	2.0	1.0	825	602	241	3.4	2.5	1.0	Yes	Yes	Yes
*Neospora caninum*	45.2	1694	1965	1695	1.0	1.2	1.0	3603	3899	2172	1.7	1.8	1.0	No	Yes	Yes
*Plasmodium falciparum*	80.6	1405	2494	1409	1.0	1.8	1.0	1938	1385	677	2.9	2.0	1.0	Yes	Yes	Yes
*Plasmodium knowlesi*	62.5	1320	2225	1330	1.0	1.7	1.0	2162	1592	736	2.9	2.2	1.0	Yes	Yes	Yes
*Plasmodium vivax*	57.7	982	1668	944	1.0	1.8	1.0	1956	1434	643	3.0	2.2	1.0	Yes	Yes	Yes
*Plasmodium yoelli*	77.4	693	2679	1338	0.5	2.0	1.0	1192	578	582	2.0	1.0	1.0	Yes	Yes	Yes
*Saccharomyces cerevisiae*	61.7	1424	2726	1498	1.0	1.8	1.0	485	391	238	2.0	1.6	1.0	Yes	Yes	Yes
*Theileria annulata*	67.5	869	1856	857	1.0	2.2	1.0	439	277	125	3.5	2.2	1.0	Yes	Yes	Yes
*Toxoplasma gondii*	47.7	1134	1878	1121	1.0	1.7	1.0	2576	2437	1623	1.6	1.5	1.0	No	Yes	Yes
*Theilera parva*	65.9	886	2052	862	1.0	2.4	1.0	376	256	154	2.4	1.7	1.0	Yes	Yes	Yes

^1^AT content of the whole genome. ^2^ In all ratios, the value for type C IGR is taken as 1. ^3^ ANOVA test with significant difference (p < 0.05) between different IGR determined using Dunn’s multiple comparison post-test.

**Figure 2:**
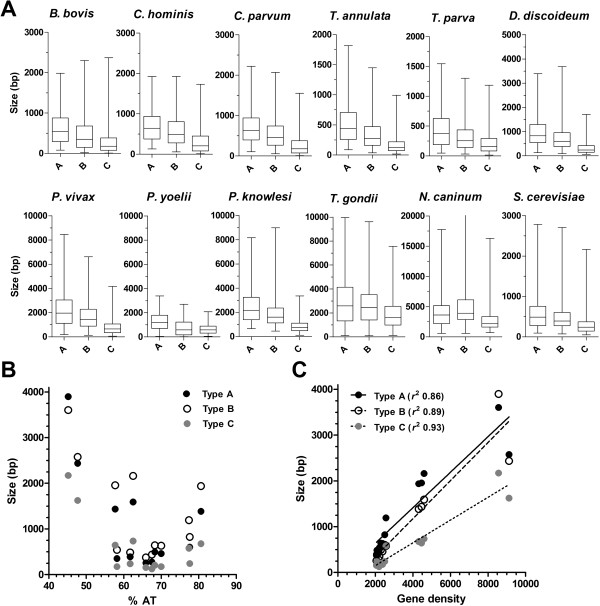
**Distribution of IGR size in 12 additional organisms. A**) Box and whisker plots representing the distribution of sizes of the different IGR types in the indicated species. Due to the distribution of data, outliers beyond 2.5-97.5% of data are not shown. See Table 2 for details relating to significance of intertype comparisons. **B**) and **C**) The median size of IGR types A, B and C for all 13 organisms plotted against the mean genomic AT content (**B**) and mean gene density (**C**).

All types of IGR show a range of median sizes across the 13 organisms investigated (Figure 2A). For all organisms where A> B > C, and all comparisons were significant (Table 2), an apparent 3:2:1 relationship is maintained in these moderately compact genomes (here 2.3-4.6 Kb/ORF) irrespective of the AT content of their genomes. Interestingly, only the two coccidian parasites, *Toxoplasma gondii* and *Neospora caninum*, do not share this same relationship, where instead A = B > C, and gene density is greatly reduced (9.1 and 8.5 Kb/ORF, respectively). Whilst no apparent relationship exists between the median sizes of the different types of IGR and the AT content (Figure 2B), there is, as expected, a strong relationship (*R*^2^ between 0.86-0.93) with the genome density, i.e. more compact genomes have proportionally smaller IGR (Figure 2C).

### *P*. *falciparum* transcripts contain a long UTR that is preferentially apportioned to the 5′ end of the ORF

**Figure 3:**
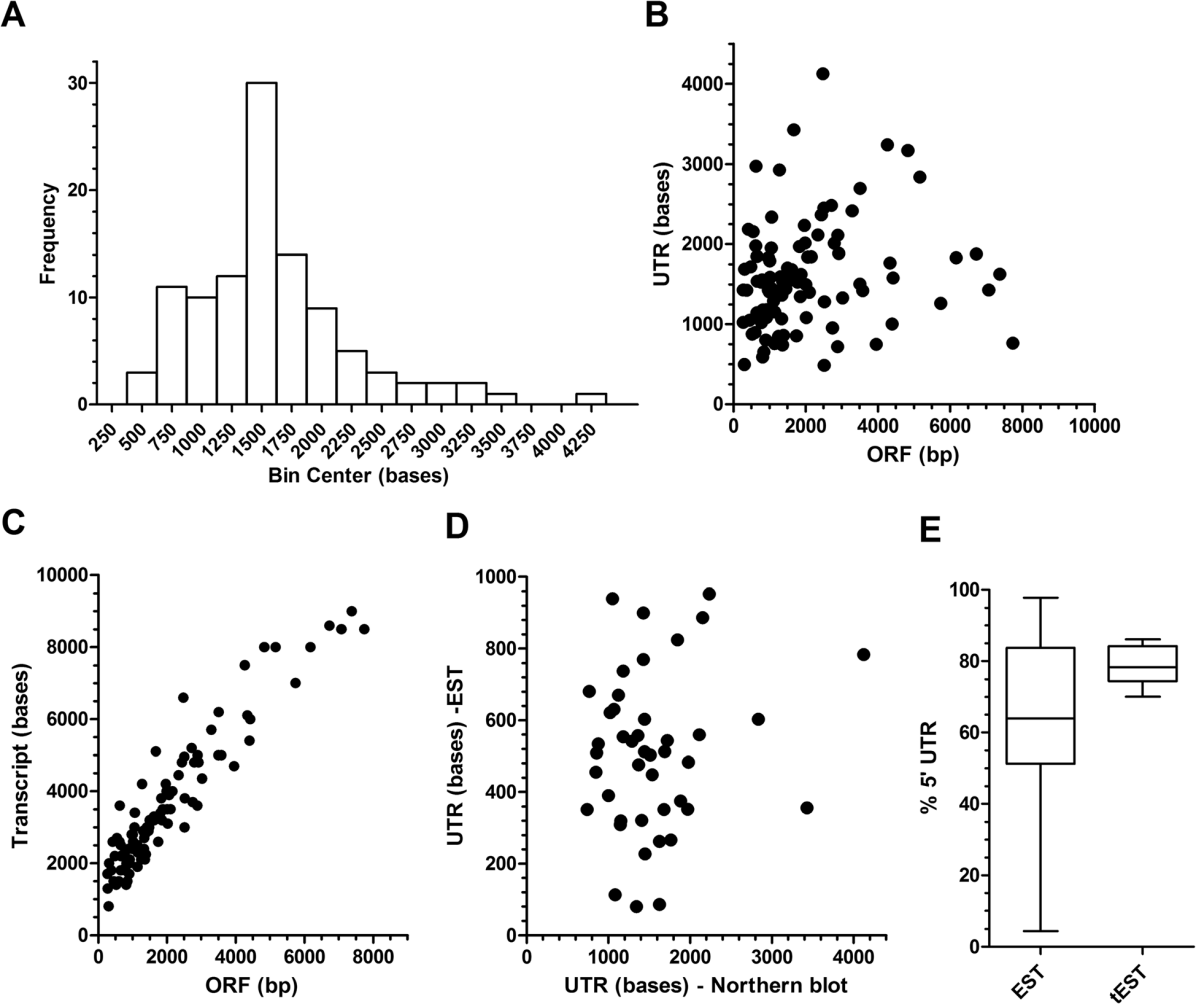
**UTR size and apportionment in *P. falciparum. *A**) Distribution of UTR sizes predicted from cohort of Northern blot data for 105 genes (bin size 250 bp). **B**) and **C**) Scatterplots comparing the size of ORF against the size of predicted UTR (B, *R*^2^ = 0.04) and the full length transcript (C, *R*^2^ = 0.88) for this cohort of genes. **D**) Scatterplot comparing the UTR sizes predicted from Northern blots and EST database sources. Only genes for which both 5′ and 3′ EST data was available are plotted (n = 44, *R*^2^ = 0.02). **E**) Box and whisker plot representing predicted apportionment to the 5′ UTR. The two plots represent either an analysis of EST data alone (n = 44 genes, EST) or a triage of this dataset (tEST). The tEST dataset (n = 19) represents only those 3′ EST that terminate with a consensus polyadenylation site.

To better understand the relationship between ORF and transcript size in *P*. *falciparum*, we collected a cohort of Northern blot data from 105 ORF. Of these, 62 were gathered during a review of the published literature with the remaining 43 from Northern blots carried out for this and other studies in our laboratory (Additional file 1 and Additional file 2). The size of the predicted UTR from these 105 transcripts revealed a diverse distribution between 486 and 4125 bases (Figure 3A, median 1518, interquartile range 1150–1844 bases). There was insufficient data to demonstrate a normal distribution, although there is clearly an evolving pattern of mono-modal distribution with 72% of all UTR sizes falling between 800–1800 bases. Comparing UTR size against the size of their respective ORF reveals no significant correlation (Figure 3B, *R*^2^ = 0.04). Given the apparent restricted distribution of the majority of UTR size, it was not surprising to find a strong correlation between the sizes of the ORF and the whole transcript (Figure 3C, *R*^2^ = 0.88), with a slope close to one (1.07 ± 0.04) and a y-intercept of 1444 ± 99 bases (close to the median distribution of 1518 bases). Sorting of the Northern blot data according to a range of criteria relating to its source, the organisation of the ORF (number of exons and orientation with respect to adjacent genes) and the morphological stage in which the peak of steady-state transcription occurs reveals no significant differences between the correlation, slope and y-intercept when comparing transcript against ORF size (Additional file 4).

Of these 105 genes, both 5′ and 3′ EST data are available for 44 (Additional file 5). The most distal 5′ and 3′ EST coordinates were secured and used together to predict a maximal UTR size. The distribution of sizes of these UTR was more restricted (range 80–952, median 512, interquartile range 351–630 bases) than those predicted from Northern blots. Notably, the sizes of the UTR from EST data were always smaller (Figure 3D) and the lack of correlation (*R*^2^ = 0.02) with UTR sizes predicted from Northern blots suggests there is unlikely to be a systematic basis to the discrepancy in size determined from the two techniques employed.

Comparison of the 5′ and 3′ EST UTR data revealed a bias in apportionment to the 5′ UTR (Figure 3E, median 61.6, range 4.8-97.8%). However, given the discrepancy between the Northern blot and EST UTR data, some caution must be applied to this provisional analysis. In order to better refine UTR apportionment, we triaged the 3′ EST sequence data (termed tEST) to identify those that contained a consensus canonical polyadenylation site motif that *P*. *falciparum* shares with other eukaryotes [37,56-59]. Of the 44 3′ EST available, 19 were identified with the remainder generally appearing to result from mis-priming of 3′ RACE from homopolymeric adenosine tracts commonly found in these AT-rich IGR. Taking the size of these 3′ UTR (range 177 to 473 bases) as a proportion of the total UTR available from Northern blots provides a more discreet set of apportionment data (Figure 3E) with a median 5′ UTR apportionment of 78.2% (range 70–86.1%).

### Modelling spatial transcript organisation over IGR

**Figure 4:**
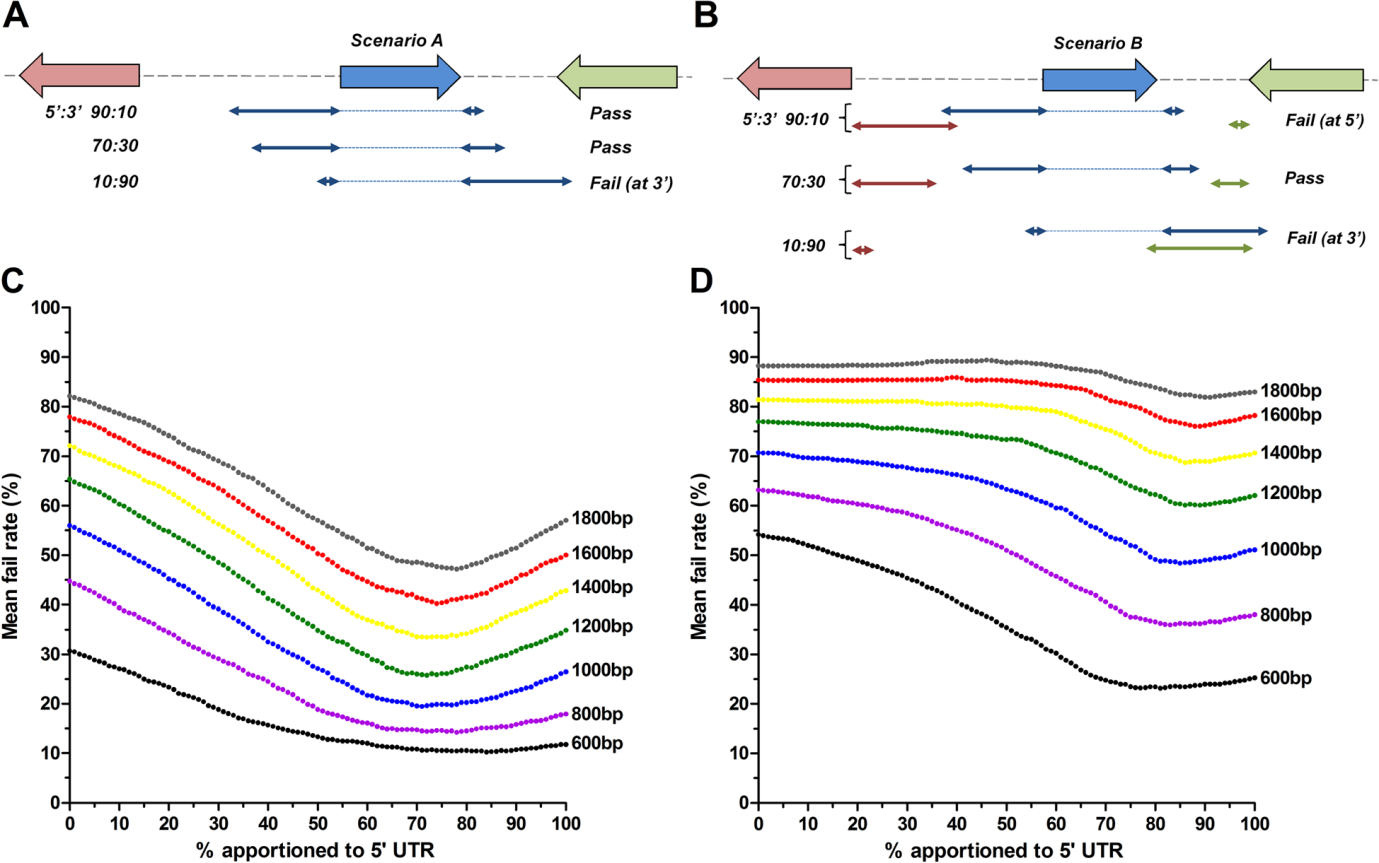
**Modelling of the spatial arrangement of UTR over IGR space in *P. falciparum*.** Schematics representing the two scenarios explored in this analysis are shown. In **A**) the UTR (double-headed arrows) apportioned over the central blue ORF (block arrow), need only fit in the total IGR space available on either side of this ORF. The apportionment is considered to fail only when the apportioned UTR overlaps with either adjacent ORF (eg. 10:90% transcript apportionment). In **B**) Scenario B is shown. Here the UTR for the blue ORF needs to fit into the IGR space available on either side of the ORF without overlapping with a similarly apportioned UTR over either flanking ORF (green and red block arrows). Examples of different apportionments of UTR are indicated to represent pass and fail. **C**) and **D**) Plots of the mean fail rate for the apportioned UTR (represented here as % apportioned to 5′ UTR) for all genes, using the indicated sizes of fixed length UTR, for Scenario A (**C**) and Scenario B (**D**).

Our data would suggest that transcripts extend further into IGR than has currently been predicted from EST and RNAseq studies. In order to explore the spatial arrangement of transcripts in the IGR flanking each ORF, in the absence of extensive mRNA coverage data, we developed a modelling approach. The aims of the modelling were to; (i) extend the evidence base for the apparent preferential 5′ UTR apportionment and (ii) explore whether transcriptional units are discrete non-overlapping entities or whether they likely overlap given the apparent large size of UTR in the relatively compact *P*. *falciparum* genome. The modelling was performed by incrementally apportioning UTR (from 100% at the 5′ to 100% at the 3′) of varying size over the IGR available around each ORF in the genome. For each ORF, length of UTR and % apportionment, a binary pass/fail was recorded – with the mean fail rate across all ORF plotted against transcript apportionment. Two scenarios were explored. The first, scenario A, considers the transcript organisation over an ORF independent of transcripts organised over adjacent ORF (Figure 4A). Thus, the UTR to be apportioned need only fit in the total IGR surrounding the ORF in question, and the tested apportionment is considered to fail only when the transcript overlaps with an adjacent ORF. This model therefore assumes that transcripts initiate and terminate solely within IGR. This was regarded as the least constrained scenario as it does not consider the nature of the adjacent transcriptional units. A second, more constrained, scenario B (Figure 4B) explores the potential for more than one transcript arrayed over an IGR; here a fail occurs when the UTR apportioned over the ORF in question overlaps with a similarly apportioned transcript over either adjacent ORF. This model therefore tests the assumption that transcripts arrayed over an IGR exist as similarly-apportioned non-overlapping entities.

Modelling of both scenarios utilised a range of fixed length UTR between 0.6 and 1.8 kb in 200 bp increments, reflecting the distribution of the majority of UTR determined above. Modelling of scenario A essentially describes a series of similarly shaped curves that show the expected inverse relationship between minimum fail rate (indicated by the lowest point on the curve) and length of UTR (Figure 4C). For all UTR lengths investigated, the best-fit was achieved when 70-80% of the UTR is apportioned to the 5′ end, correlating well with the triaged EST UTR data described above (70–86.1% at 5′ end). Similarly, using the more constrained scenario B, for all UTR lengths investigated the best-fit is achieved when the majority of UTR is apportioned to the 5′ end, although here there is a slight increase to a 75-85% 5′ apportionment (Figure 4D). The key difference between the two scenarios is the significant increase in fail rates obtained, irrespective of the length of UTR modelled, when attempting to fit two non-overlapping transcripts over the IGR space available. Minimum fail rates that range between 10.2 and 47.8% in scenario A increase dramatically to between 23.2 and 81.8% in scenario B (values represent minimum fail rates for 600 and 1800 bases UTR). Our modelling suggests that the assumption that transcripts are arrayed over an IGR as non-overlapping entities is incorrect. Moreover, the high fail rates in scenario A suggest that the second assumption that transcriptional start and stop sites are solely located within IGR may similarly not be true. However, it is worth noting these are mean fail rates and the data can be granulated accordingly to determine the effects of different possible orientations of types of flanking sequence around an ORF. As expected, ORF with large amounts of flanking sequence (type A at 5′ and B at 3′) have lower fail rates, with the corresponding opposite effect where less flanking sequence (type B at 5′ and C at 3′) is available (data not shown). Whilst the potential for smaller transcripts apportioned over ORF with smaller IGR spaces around them is possible – examination of the UTR size for the different orientations of the 105 genes in the Northern blot cohort data revealed no significant difference on this basis (Additional file 4).

### Temporal organisation of transcription over IGR during the intraerythrocytic development cycle

**Figure 5:**
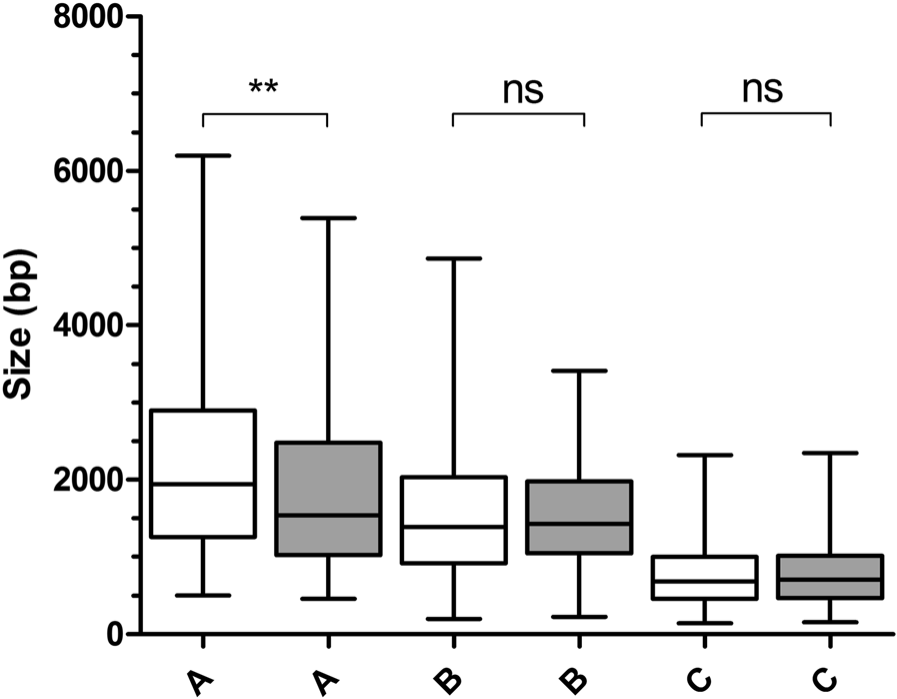
**Analysis of temporal co-transcription on IGR size in *P. falciparum. ***Clear boxes represent the distribution of sizes for all of the indicated IGR type, with the grey shaded boxes representing the distribution of IGR sizes over which two transcripts occur within the same temporal window during intraerythrocytic development. For each IGR type, the result of an ANOVA test is shown (ns, not significant, ** p < 0.01 in Dunn’s multiple comparison post-test). Due to the distribution of data, outliers beyond the 2.5-97.5% of data represented by the range whiskers are not shown.

Our modelling suggests that there is likely a significant programme of transcriptional overlap within IGR. The premise that two transcripts are necessarily synthesised simultaneously over both template strands of an IGR, however, may not generally occur given the extensive programme of stage-specific transcription that occurs during the parasite’s progression through its complex life cycle [10,60,61]. We therefore explored the potential for co-spatial and co-temporal transcription over the IGR that flank the 3835 ORF that are transcribed during the intraerythrocytic development cycle (IDC). Comprehensive stage-specific transcriptomic datasets are available and provide an opportunity to define peak transcript accumulation to defined temporal windows of the 46-48 hr IDC [10-12,62,63]. We adopted the organisation of these 3835 ORF into four clusters described by Jurgelenaite and colleagues [62]. Each cluster represents a group of temporally co-transcribed genes, with peaks of steady state transcript levels in the following morphologically distinguishable intraerythrocytic developmental stages (i) early ring, (ii) late ring and early trophozoite, (iii) trophozoite and schizont, and (iv) schizont only stages. Of the total of 5588 IGR, only 568 (10.2%) shared transcripts from both flanking ORF within the same window of peak temporal transcription during the IDC. Specifically, these were; 202 type A (13.7% of total type A), 237 type B (9%) and 129 type C (8.7%), with type A IGR appearing slightly overrepresented in this analysis. Comparison of the median sizes of these co-transcribed IGR still show that the A > B > C relationship holds true (Figure 5, median sizes of 1539, 1428 and 705 bp, respectively). However, whilst the sizes of types B and C cotranscribed IGRs are not significantly different from those in the whole genome, those of cotranscribed type A IGR are significantly smaller (Figure 5). We note that whilst a total of 10.2% of spatially overlapping transcripts in *P*. *falciparum* is similar to that determined in *S*. *cerevisiae* and other eukaryotes, this value is probably an overestimate given the relatively broad windows of time used to define co-temporal transcription (8-12 hrs) in this analysis.

## Discussion

This study set out to address a fundamental gap in our understanding of the *P*. *falciparum* transcriptional unit outside of the ORF. Specifically, we examined the size and apportionment of the UTR as well as the spatial and temporal organization of the transcriptional units within the IGR that flank these ORF. In terms of the size and apportionment of UTR, our data would indicate; (i) that UTR are long, typically some 800–1800 bases, (ii) that the size of the UTR is independent of the size of the coding sequence and (iii) that 70-80% of the UTR is preferentially apportioned 5′ of the ORF. This would indicate that transcriptional start and stop sites lay between 600-1350 bp and 200-450 bp either side of the ORF. Apart from lengthening our current understanding of the extent of the transcriptional landscape in *P*. *falciparum*, these more distal transcriptional coordinates have implications for our search and validation of regulatory *cis*-acting regions. *In silico* searches for sequence motifs enriched in the flanking regions of functionally related and/or cotranscribed genes typically use 1kbp of flanking sequence [64,65]. Whilst this would seem suitable for searching downstream of an ORF, it is perhaps not sufficient to identify all potential 5′ positioned regulatory elements. That said, a ScanACE analysis of at least 2kbp of flanking sequence has provided an extensive catalogue of putative ApiAP2 transcription factor binding sites [22]. Testing of these putative sites will require functional analyses of promoter activity. Our data regarding the extent of UTR coverage, as well as the significant chance of transcript overlap, provides insights that may help guide selection of sites more likely to be *trans*-acting factor binding sites to be tested in these studies.

Of note was the discrepancy between the sizes of UTR predicted from Northern blot and EST data; with those predicted from EST data invariably being shorter. This discrepancy is unlikely to result from a selection bias in the cohort of 105 genes used in this study as the mean size of all 5′ UTR from the EST data for these genes (305 ± 182 bp) is very similar to that published for 1465 genes for which 5′ EST data is available (303 ± 155 bp) [31]. More likely, bias introduced into the EST data by; (i) reduced processivity of reverse transcriptase over AT rich sequences, (ii) partial RNAseH activity in early generation enzymes and (iii) the use of oligo(dT) for first strand cDNA synthesis in some EST datasets, are all at play. Northern blot data are similarly prone to systematic error as often these are “guestimates” based on the use of a limited set of size standards during electrophoretic size fractionation. We also recognise the limitations arising from analysis of 105 genes by Northern blot analysis (*c*. 2% of all genes). This study does, however, represent the most complete meta-analysis of Northern blot data in *P*. *falciparum* to date.

Assuming a range of UTR between 800 and 1800 bases would indicate that 40-90% of all IGR space in the relatively compact genome of *P*. *falciparum* is included in at least one transcript. Since it would appear likely that there is significant transcriptional unit overlap, the actual extent of this transcriptional landscape over the genome would be reduced, although our data would suggest it is still considerably more than previously predicted from the available RNAseq and EST coverage. Why these UTR are so large in *P*. *falciparum* is intriguing. The size of the UTR, in part, would require that it is long enough to contain the *cis*-regulatory elements necessary for RNA metabolism. Whilst we know relatively little about these, the high level of selective constraint throughout intergenic regions in *P*. *falciparum* provides evidence of an evolutionary “footprint” for these non-coding elements [66,67]. Selective constraint is slightly, although not significantly, higher in proximal intergenic regions [66], i.e. regions more likely encoded in the UTR. In itself, however, the presence of these *cis*-regulatory elements doesn’t provide an explanation for the length of the UTR. The extreme AT bias of these IGR, however, may provide some explanation for this phenomenon. Like *P*. *falciparum*, transcripts in *D*. *discoidium* have long UTR with a median length of 724 bp for the 14124 5′ UTR sequences deposited in Dictybase. Both organisms share a highly biased AT-rich genome, effectively resulting in a binary nucleotide code within the IGR. This reduction in information content may necessarily lead to an expansion of sequences necessary to encode/utilise regulatory information, although this is perhaps an oversimplified interpretation of the observation. Critically, the genomes of both organisms show evidence of extensive overrepresentation of homopolymeric poly(dA).poly(dT) tracks [68,69], and these tracts are more highly overrepresented within the IGR (own unpublished data). Thus, a requirement to maintain non-coding *cis*-regulatory elements embedded within flexible poly(dA).(dT) tracts that are prone to expansion could account for the increased length of UTR in *P*. *falciparum*. This proposal would suggest that some regions within the UTR are less essential than others - an observation borne out by our own (Hasenkamp S, Russell K, Ullah I, Horrocks P: Functional analysis of the 5′ untranslated region of the phosphoglutamase 2 transcript in *Plasmodium falciparum*, *in press*) and other studies that have determined the effect on reporter gene expression following deletion of UTR sequences [70-73]. Deletions of several hundred bases of the proximal 5′UTR appear to have a minimal effect on the absolute and temporal expression of the reporter gene, suggesting some plasticity in the size of the *P*. *falciparum* transcript.

Our analysis of IGR organisation in *P*. *falciparum* would indicate; (i) that the observed 1:1.8:1 relationship for IGR types A, B and C, respectively, is close to the predicted 1:2:1 ratio expected of independently-organised monocistronic transcriptional units and (ii) that IGR size directly correlates with the nature of the transcriptional activity that occur over them with a ratio of 2.86:2.05:1. Szafranski *et al*., using partial genome sequence from *S*. *cerevisiae*, *D*. *discoidium*, *A*. *thaliana* and *P*. *falciparum*, reported a provisional investigation of features of AT-rich organisms that may assist in genome annotation [29]. In doing so, they predicted that relatively compact genomes would share a 3:2:1 gene spacing rule for IGR types A, B and C. Their study couldn’t correlate this 3:2:1 rule to AT content due to the limited diversity of organisms investigated. Here we have extended this analysis of IGR to encompass the entire genomes of 13 organisms, exhibiting a range of AT content and genome density, albeit with a focus on other apicomplexan parasites. In this larger study, we confirm that IGR size does not correlate with AT content, whereas we do find, perhaps not unexpectedly, that IGR size does correlate with the overall genome density, with a close linear relationship (*R*^2^ between 0.84-0.98) for genome densities between 2.3-4.6 Kb/ORF. This correlation, although weaker does extend out to the 9.1 Kb/ORF gene density found in *T*. *gondii*, although here the 3:2:1 gene spacing rule apparently collapses to an approximate 1.5:1.5:1 ratio. A novel finding in this study, however, was the differing spatial arrangement of IGR size within different chromosomal compartments in *P*. *falciparum*, where IGR lengths, irrespective of their type, are longer in subtelomeric regions. Multigene families that encode proteins likely to mediate interactions with the host environment are preferentially located in this compartment and are best exemplified by the *var* family that encodes the *P*. *falciparum* erythrocyte membrane protein (PfEMP1) [9,41,46]. PfEMP1 are exposed on the surface of infected erythrocytes where they mediate adhesion to host cell surface ligands and, through clonal variation of the PfEMP1 expressed, help to establish a chronic infection in the face of a human immune response mounted against infected erythrocytes. We would speculate that this immune response may act a balancing selection pressure to that operating in the chromosomal internal compartment to reduce gene density through reduction in IGR size [74]. Repetitive sequence elements within the longer IGR in subtelomeric regions may assist in the organisation of chromosome ends at the nuclear periphery, a necessary factor in the epigenetic regulation of clonal expression, or may promote recombination to drive the generation of antigenic diversity in these multigene families.

## Conclusions

Taken together, our data provides a theoretical framework for the spatial and temporal organisation of transcripts over the IGR, data that are not available from current microarray, EST and RNAseq analyses. With the potential for the next generation of directional RNAseq data to extend cDNA coverage into the IGR, we propose here a series of testable hypotheses that result from our theoretical framework. Specifically, we would predict; (i) UTR are typically between 800 and 1800 bp in size, (ii) 70-80% of UTR are preferentially organised to the 5’ of the transcript, (iii) 40-90% of the IGR sequences are transcribed, resulting in 70-80% of the entire genome organised within a transcript, (iv) that whilst UTR do not temporally overlap, a significant proportion will spatially overlap and (v) that a small number (up to 200) of bidirectional promoters exist. In addition, our findings suggest that how we think about the transcriptional landscape across the *P*. *falciparum* genome should be revised to a view that is more dynamic in terms of direction, timing and extent of coverage of transcription over the genomic template. These insights should impact on how we design studies to define and characterise functional elements that govern processes such as developmentally-linked gene expression and monoallelic expression of virulence-linked multigene families. Finally, since we show the organisation of IGR in related apicomplexans appears to follow the same spatial rules, aspects of this work may translate more widely across this group of parasites important to human and veterinary health.

## Methods

### Cohort of Northern blot data

Transcript sizes for 43 genes were available as unpublished data from our laboratory. These were generated using the same general method as previously described [75]. Northern blots of total cellular RNA were prepared and hybridized at 50°C with 500-800 bp DNA fragments obtained from PCR over single introns of genes of interest, labelled with alpha-^32^P-dATP using Megaprime (GE Healthcare/Amersham Bioscience), and exposed for 8-48 hrs and the image processed using a Cyclone storage phosphor screen apparatus controlled using OptiQuant software (Packard). The remaining 62 transcript sizes were determined from a review of the published literature. Criteria for inclusion in this study were; (i) the manuscript had to specifically state the size of the transcript or (ii) show a figure of the transcript with size markers to enable an estimate to be made and (iii) not be a member of a multigene family (often cannot reliably allocate transcript to specific ORF).

### Capture of IGR size and orientation

General feature format (GFF) files were obtained for each of the organisms (where available, strain/isolate/clone indicated) investigated. These were obtained from; Genbank (*B*. *bovis* Texas T2Bo, *T*. *parva* Mugugu, *T*. *annulata* Ankara clone C9), CryptoDB 4.0 (*C*. *hominis* Tu502, *C*. *parvum* Iowa), DictyBase (*D*. *discoideum*), ToxoDB 5.1 (*N*. *caninum* Liverpool, *T*. *gondii* ME49), PlasmoDB 5.5 (*P*. *falciparum* 3D7, *P*. *knowlesi* H strain, *P*. *vivax* Salvador I, *P*. *yoelii* 17XNL) and Saccharomyces Genome DB (*S*. *cerevisiae*). Using the start/end coordinates and strand orientation fields, the size of each IGR and the orientation of the flanking ORF were determined with the latter used to categorise these IGR into three types (A-C) as described in the results section of the manuscript. Analysis of the distribution of the size of these types of IGR was by a Kruskal–Wallis one-way analysis of variance (ANOVA) with a Dunn’s multiple comparison post-test (GraphPad Prism v5.01, USA).

### Correlation of IGR size with microarray datasets

Jurgelenaite et al. reports an analysis of the IDC transcription profiles of 3835 ORF, producing 5 clusters of genes that exhibit either a shared temporal peak of transcription (4 clusters) or share an apparent constitutive pattern of transcription throughout the IDC [62]. The 2491 ORF listed within the 4 temporal windows of transcription were parsed against the lists of pairs of genes that flank each IGR. Those IGR for which both genes share the same temporal window of transcription were secured and categorised into types A-C and the distribution of the size of these IGR analysed as described above.

### Modelling apportionment of the UTR

Using the GFF annotation file for *P*. *falciparum* 3D7 the start/stop coordinates for each ORF and both upstream and downstream flanking genes were determined. From these data the size of each flanking IGR was calculated. A length of UTR (fixed increments of 200 bp for whole genome or actual size of UTR for cohort of 105 genes used here) was sequentially apportioned in 1% increments from 100% at the 5′ of the ORF to 100% at the 3′. Overlap of the UTR with flanking ORF (Scenario A) or with a similarly apportioned UTR allocated to both flanking ORF (Scenario B) was recorded as a failed apportionment. A set of Perl language scripts were developed to automate these tasks and are available at http://sites.google.com/site/emesbioinformatics/group-software.

## Competing interests

The authors declare they have no competing interests.

## Authors’ contributions

KR carried out the Northern blot studies, analysed the IGR and modelling data and drafted the initial manuscript. SH carried out the Northern blot studies and analysed UTR apportionment data. RE designed and wrote the algorithms used in the study and assisted in analysing the modelling data. PH designed the study, helped design the modelling algorithms, analysed the data and coordinated the production of the final manuscript. All authors have read and approved the final manuscript.

## Supplementary Material

Additional file 1 **Cohort of 105 ORF from *P. falciparum *for which Northern blot data was collated.** Click here for file

Additional file 2 **Reference list for Additional file ****2.** Click here for file

Additional file 3 **Breakpoints used to define chromosomal compartments in *P. falciparum.*** Click here for file

Additional file 4 Extended regression analysis of cohort of Northern blot data. Click here for file

Additional file 5 Comparison of Northern blot and EST data. Click here for file
