# Supplementary material for: Analysis of the spatial and temporal arrangement of transcripts over intergenic regions in the human malarial parasite Plasmodium falciparum
Source: BMC Genomics. 2013 Apr 19;14:267. doi: 10.1186/1471-2164-14-267 (PMC3681616; doi:10.1186/1471-2164-14-267)
Supplement: Additional file 2 — Reference list for Additional file 2. [file 1471-2164-14-267-S2.docx]

**Additional file 2:** Reference list for additional file 2

Alano *et al.* (1996) Structure and polymorphism of the upstream region of the *pfg27/25* gene, transcriptionally regulated in gametocytogenesis of *Plasmodium falciparum*. *Mol Biochem Parasitol,* 79, 207-217.

Balu *et al.* (2009) Identification of the transcription initiation site reveals a novel transcript structure for *Plasmodium falciparum maebl*. *Exp Parasitol,* 121, 110-114.

Barik *et al.* (1997) Identification, cloning, and mutational analysis of the casein kinase 1 cDNA of the malaria parasite, *Plasmodium falciparum*: stage-specific expression of the gene. *J Biol Chem,* 272, 26132-8.

Berry C*, et al.* (1999) A distinct member of the aspartic proteinase gene family from the human malaria parasite *Plasmodium falciparum*. *FEBS Lett,* 447, 149-54.

Bracchi-Ricard *et al.* (2000) PfPK6, a novel cyclin-dependent kinase/mitogen-activated protein kinase-related protein kinase from *Plasmodium falciparum*. *Biochem J,* 347, 255-63.

Cheesman *et al.* (1998) Intraerythrocytic expression of topoisomerase II from *Plasmodium falciparum* is developmentally regulated. *Mol Biochem Parasitol,* 92, 39-46.

Delves *et al.* (1990) Expression of alpha and beta tubulin genes during the asexual and sexual blood stages of *Plasmodium falciparum*. *Mol Biochem Parasitol,* 43, 271-8.

Dobson *et al.* (2001) Characterization of a novel serine/threonine protein phosphatase (PfPPJ) from the malaria parasite, *Plasmodium falciparum*. *Mol Biochem Parasitol,* 115, 29-39.

Dobson *et al.* (2003) Characterization of a unique aspartate-rich protein of the SET/TAF-family in the human malaria parasite, *Plasmodium falciparum*, which inhibits protein phosphatase 2A. *Mol Biochem Parasitol,* 126, 239-50.

Doerig *et al.* (1995) Pfcrk-1, a developmentally regulated cdc2-related protein kinase of *Plasmodium falciparum*. *Mol Biochem Parasitol,* 70, 167-74.

Doerig *et al.* (1996) A MAP kinase homologue from the human malaria parasite, *Plasmodium falciparum*. *Gene,* 177, 1-6.

Fan *et al.* (2004a) *Plasmodium falciparum* Histone Acetyltransferase, a Yeast GCN5 Homologue Involved in Chromatin Remodeling. *Euk Cell,* 3, 264-276.

Fan *et al.* (2004b) PfADA2, a *Plasmodium falciparum* homologue of the transcriptional coactivator ADA2 and its *in vivo* association with the histone acetyltransferase PfGCN5. *Gene,* 336, 251-261.

Foote *et al.* (1989) Amplification of the multidrug resistance gene in some chloroquine-resistant isolates of *P. falciparum*. *Cell,* 57, 921-930.

Fox & Bzik (1991) The primary structure of *Plasmodium falciparum* DNA polymerase delta is similar to drug sensitive delta-like viral DNA polymerases. *Mol Biochem Parasitol,* 49, 289-96.

Hansen *et al.* (2002) A single, bi-functional aquaglyceroporin in blood-stage *Plasmodium falciparum* malaria parasites. *J Biol Chem,* 277, 4874-82.

Hicks *et al.* (1991) Glycolytic pathway of the human malaria parasite *Plasmodium falciparum*: primary sequence analysis of the gene encoding 3-phosphoglycerate kinase and chromosomal mapping studies. *Gene,* 100, 123-9.

Holloway *et al.* (1994) Isolation and characterization of a chaperonin-60 gene of the human malaria parasite *Plasmodium falciparum*. *Mol Biochem Parasitol,* 64, 25-32.

Horrocks *et al.* (1996) Stage specific expression of proliferating cell nuclear antigen and DNA polymerase delta from *Plasmodium falciparum*. *Mol Biochem Parasitol,* 79, 177-182.

Horrocks & Kilbey (1996) Physical and functional mapping of the transcriptional start sites of *Plasmodium falciparum* proliferating cell nuclear antigen. *Mol Biochem Parasitol,* 82, 207-215.

Horrocks & Newbold (2000) Intraerythrocytic polyubiquitin expression in *Plasmodium falciparum* is subjected to developmental and heat-shock control. *Mol Biochem Parasitol,* 105, 115-125.

Horrocks *et al.* (2002) Stage-specific promoter activity from stably maintained episomes in *Plasmodium falciparum*. *Int J Parasitol,* 32, 1203-1206.

Joshi *et al.* (1999) Molecular cloning and nuclear localization of a histone deacetylase homologue in *Plasmodium falciparum*. *Mol Biochem Parasitol,* 99, 11-19.

Knapp *et al.* (1990) *Plasmodium falciparum* aldolase: gene structure and localization. *Mol Biochem Parasitol,* 40, 1-12.

Krnajski *et al.* (2002) Thioredoxin reductase is essential for the survival of *Plasmodium falciparum* erythrocytic stages. *J Biol Chem,* 277, 25970-5.

Kyes *et al.* (2002) Stage-specific merozoite surface protein 2 antisense transcripts in *Plasmodium falciparum*. *Mol Biochem Parasitol,* 123, 79-83.

Kyes *et al.* (2000) A simple RNA analysis method shows *var* and *rif* multigene family expression patterns in *Plasmodium falciparum*. *Mol Biochem Parasitol,* 105, 311-315.

Lanzer *et al.* (1992a) Transcription mapping of a 100 kb locus of *Plasmodium falciparum* identifies an intergenic region in which transcription terminates and reinitiates. *EMBO J,* 11, 1949-55.

Lanzer *et al.* (1992b) A sequence element associated with the *Plasmodium falciparum* *kahrp* gene is the site of developmentally regulated protein-DNA interactions. *Nuc Acids Res,* 20, 3051-3056.

Lanzer *et al.* (1994) Transcriptional and nucleosomal characterization of a subtelomeric gene-cluster flanking a site of chromosomal rearrangements in *Plasmodium falciparum*. *Nuc Acids Res,* 22, 4176-4182.

Li *et al.* (2004) Isolation and functional characterization of a dynamin-like gene from *Plasmodium falciparum*. *Biochem Biophys Res Commun,* 320, 664-71.

Li *et al.* (2002) Identification of a second proliferating cell nuclear antigen in the human malarial pathogen *Plasmodium falciparum*. *Int J Parasitol,* 32, 1683-1692.

Li *et al.* (1989) An enlarged largest subunit of *Plasmodium falciparum* RNA polymerase II defines conserved and variable RNA polymerase domains. *Nuc Acids Res,* 17, 9621-36.

Li *et al.* (1991) Characterization of the gene encoding the largest subunit of *Plasmodium falciparum* RNA polymerase III. *Mol Biochem Parasitol,* 46, 229-39.

Martin *et al.* (2000) Characterization of *Plasmodium falciparum* CDP-diacylglycerol synthase, a proteolytically cleaved enzyme. *Mol Biochem Parasitol,* 110, 93-105.

Mello *et al.* (2002) A multigene family that interacts with the amino terminus of *Plasmodium* MSP-1 identified using the yeast two-hybrid system. *Euk Cell,* 1, 915-925.

Myrick *et al.* (2003) Mapping of the *Plasmodium falciparum* multidrug resistance gene 5' upstream region, and evidence of induction of transcript levels by antimalarial drugs in chloroquine sensitive parasites. *Mol Microbiol,* 49, 671-683.

Olafsson *et al.* (1992) Molecular analysis of *Plasmodium falciparum* hexokinase. *Mol Biochem Parasitol,* 56, 89-101.

Osta *et al.* (2002) A 24bp *cis*-acting element essential for the transcriptional activity of *Plasmodium falciparum* CDP-diacylglycerol synthase gene promoter. *Mol Biochem Parasitol,* 121, 87-98.

Patterson S*, et al.* (2002) Molecular characterization and expression of an alternate proliferating cell nuclear antigen homologue, PfPCNA2, in *Plasmodium falciparum*. *Biochem. Biophys. Res. Comm.,* 298, 371-376.

Prasartkaew *et al.* (1996) Molecular cloning of a *Plasmodium falciparum* gene interrupted by 15 introns encoding a functional primase 53 kDa subunit as demonstrated by expression in a baculovirus system. *Nuc Acids Res,* 24, 3934-41.

Przyborski *et al.* (2003) The histone *H4* gene of *Plasmodium falciparum* is developmentally transcribed in asexual parasites. *Parasitol Res,* 90, 387-9.

Spielmann & Beck (2000) Analysis of stage-specific transcription in *Plasmodium falciparum* reveals a set of genes exclusively transcribed in ring stage parasites. *Mol Biochem Parasitol,* 111, 453-8.

Syin & Goldman (1996) Cloning of a *Plasmodium falciparum* gene related to the human 60-kDa heat shock protein. *Mol Biochem Parasitol,* 79, 13-9.

Tosh *et al.* (1999) *Plasmodium falciparum*: stage-related expression of topoisomerase I. *Exp Parasitol,* 91, 126-32.

Tosh & Kilbey (1995) The gene encoding topoisomerase I from the human malaria parasite *Plasmodium falciparum*. *Gene,* 163, 151-154.

Volkman *et al.* (1993) Stage-specific transcripts of the *Plasmodium falciparum pfmdr-1* gene. *Mol Biochem Parasitol,* 57, 203-212.

Waller *et al.* (2003) Chloroquine resistance modulated *in vitro* by expression levels of the *Plasmodium falciparum* chloroquine resistance transporter. *J Biol Chem,* 278, 33593-601.

Watanabe (1997) Cloning and characterization of heat shock protein DnaJ homologues from *Plasmodium falciparum* and comparison with ring infected erythrocyte surface antigen. *Mol Biochem Parasitol,* 88, 253-8.

Wellems & Howard (1986) Homologous genes encode two distinct histidine-rich proteins in a cloned isolate of *Plasmodium falciparum*. *Proc Natl Acad Sci USA,* 83, 6065-9.

Wesseling *et al.* (1989) Stage-specific expression and genomic organization of the actin genes of the malaria parasite *Plasmodium falciparum*. *Mol Biochem Parasitol,* 35, 167-76.

White *et al.* (1993) The gene encoding DNA polymerase-alpha from *Plasmodium falciparum*. *Nucl Acids Res,* 21, 3643-3646.

Wickramarachchi *et al.* (2008) Identification and characterization of a novel *Plasmodium falciparum* merozoite apical protein involved in erythrocyte binding and invasion. *Plos One,* 3, e1732.

Wilson *et al.* (1989) Amplification of a gene related to mammalian *mdr* genes in drug-resistant *Plasmodium falciparum*. *Science,* 244, 1184-1186.

Zhao *et al.* (1993) Gene structure and expression of an unusual protein-kinase from *Plasmodium falciparum* homologous at its carboxyl terminus with the EF hand calcium-binding proteins. *J Biol Chem,* 268, 4347-4354.
