# Supplementary material for: Analysis of the spatial and temporal arrangement of transcripts over intergenic regions in the human malarial parasite Plasmodium falciparum
Source: BMC Genomics. 2013 Apr 19;14:267. doi: 10.1186/1471-2164-14-267 (PMC3681616; doi:10.1186/1471-2164-14-267)
Supplement: Additional file 3 — Breakpoints used to define chromosomal compartments in P. falciparum. [file 1471-2164-14-267-S3.docx]

**Additional file 3.** Breakpoints used to define chromosomal compartments in *P. falciparum.* The table lists the PlasmoDB unique identifiers for genes that were used in this study to define breakpoints between chromosomal internal and subtelomeric domains.
