# Supplementary material for: Analysis of the spatial and temporal arrangement of transcripts over intergenic regions in the human malarial parasite Plasmodium falciparum
Source: BMC Genomics. 2013 Apr 19;14:267. doi: 10.1186/1471-2164-14-267 (PMC3681616; doi:10.1186/1471-2164-14-267)
Supplement: Additional file 4 — Extended regression analysis of cohort of Northern blot data. [file 1471-2164-14-267-S4.pptx]

## Slide 1
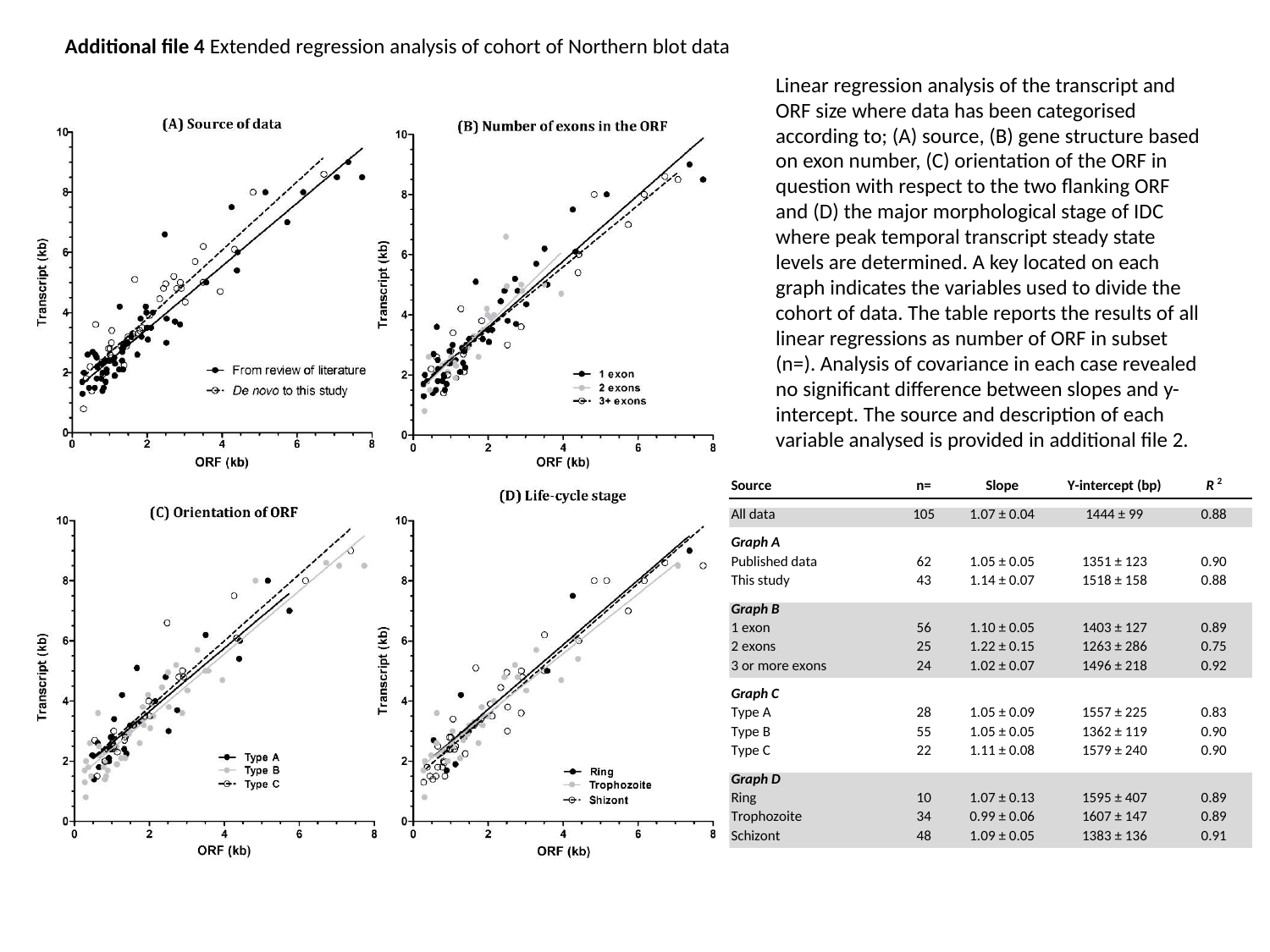

Additional file 4 Extended regression analysis of cohort of Northern blot data
Linear regression analysis of the transcript and ORF size where data has been categorised according to; (A) source, (B) gene structure based on exon number, (C) orientation of the ORF in question with respect to the two flanking ORF and (D) the major morphological stage of IDC where peak temporal transcript steady state levels are determined. A key located on each graph indicates the variables used to divide the cohort of data. The table reports the results of all linear regressions as number of ORF in subset (n=). Analysis of covariance in each case revealed no significant difference between slopes and y-intercept. The source and description of each variable analysed is provided in additional file 2.
